# Broad-spectrum antiviral activity of chebulagic acid and punicalagin against viruses that use glycosaminoglycans for entry

**DOI:** 10.1186/1471-2180-13-187

**Published:** 2013-08-07

**Authors:** Liang-Tzung Lin, Ting-Ying Chen, Song-Chow Lin, Chueh-Yao Chung, Ta-Chen Lin, Guey-Horng Wang, Robert Anderson, Chun-Ching Lin, Christopher D Richardson

**Affiliations:** 1 Department of Microbiology and Immunology, School of Medicine, College of Medicine, Taipei Medical University, Taipei, Taiwan; 2 Department of Pediatrics and Canadian Center for Vaccinology, IWK Health Centre, Halifax, Nova Scotia, Canada; 3 School of Pharmacy, College of Pharmacy, Kaohsiung Medical University, Kaohsiung, Taiwan; 4 Department of Pharmacology, School of Medicine, College of Medicine, Taipei Medical University, Taipei, Taiwan; 5 Graduate Institute of Natural Products, College of Pharmacy, Kaohsiung Medical University, Kaohsiung, Taiwan; 6 Graduate Institute of Pharmaceutical Science and Technology, Central Taiwan University of Science and Technology, Taichung, Taiwan; 7 Department of Cosmetic Science, Chia Nan University of Pharmacy and Science, Tainan, Taiwan; 8 Department of Microbiology and Immunology, Dalhousie University, Halifax, Nova Scotia, Canada

**Keywords:** Chebulagic acid, Punicalagin, Tannin, Broad-spectrum antivirals, Viral entry, Glycosaminoglycans

## Abstract

**Background:**

We previously identified two hydrolyzable tannins, chebulagic acid (CHLA) and punicalagin (PUG) that blocked herpes simplex virus type 1 (HSV-1) entry and spread. These compounds inhibited viral glycoprotein interactions with cell surface glycosaminoglycans (GAGs). Based on this property, we evaluated their antiviral efficacy against several different viruses known to employ GAGs for host cell entry.

**Results:**

Extensive analysis of the tannins’ mechanism of action was performed on a panel of viruses during the attachment and entry steps of infection. Virus-specific binding assays and the analysis of viral spread during treatment with these compounds were also conducted. CHLA and PUG were effective in abrogating infection by human cytomegalovirus (HCMV), hepatitis C virus (HCV), dengue virus (DENV), measles virus (MV), and respiratory syncytial virus (RSV), at μM concentrations and in dose-dependent manners without significant cytotoxicity. Moreover, the natural compounds inhibited viral attachment, penetration, and spread, to different degrees for each virus. Specifically, the tannins blocked all these steps of infection for HCMV, HCV, and MV, but had little effect on the post-fusion spread of DENV and RSV, which could suggest intriguing differences in the roles of GAG-interactions for these viruses.

**Conclusions:**

CHLA and PUG may be of value as broad-spectrum antivirals for limiting emerging/recurring viruses known to engage host cell GAGs for entry. Further studies testing the efficacy of these tannins *in vivo* against certain viruses are justified.

## Background

Viral infections are responsible for causing a significant number of human diseases, epidemic outbreaks, morbidity, and mortality. While vaccine efforts have proven successful for preventing and eradicating some viral infections, many viruses cannot be targeted by immunization, including dengue virus (DENV), human cytomegalovirus (HCMV), hepatitis C virus (HCV), human immunodeficiency virus (HIV), and respiratory syncytial virus (RSV) [1-5]. Alternative means of control include the use of antiviral drugs; however, there are currently few licensed and efficacious drugs available for prophylactic and therapeutic antiviral treatments. Global public health is therefore under constant threat of emerging and re-emerging viral infections, particularly those that do not currently have effective vaccines or have the potential to develop drug-resistant mutations [6]. Furthermore, due to increased global travel, trade, and rapid urbanization, increased numbers of viral pathogens are being introduced or re-introduced into areas where they are not normally indigenous [7]. This is reflected by the recent emergence of viral outbreaks caused by severe acute respiratory syndrome (SARS) virus, influenza virus (H1N1 and H5N1), DENV, West Nile virus (WNV), and measles virus (MV) [7-9]. In addition, the potential for outbreaks due to the intentional or accidental release of virus has also raised serious concerns. Thus, efforts in developing antiviral therapies are required to safeguard the public against viral pathogens.

Most antiviral therapies target defined steps in the viral life cycle, or more specifically, a particular viral protein. Examples include nucleoside analogues that inhibit herpes simplex virus (HSV) replication [10], protease inhibitors directed against the HCV NS3 protease [11], and neuraminidase inhibitors that block the release of influenza virus particles from infected cells [12]. However, the use of these antivirals is inevitably associated with the potential risk of selecting for drug-resistant viruses, which can pose a significant problem in the clinical management of these viral infections [10,12,13]. A combination cocktail of several inhibitors is often necessary to reduce the risk of generating drug resistant mutants. This is best exemplified by Highly Active Antiretroviral Therapy (HAART) for treating HIV infections [14]. However, experience with combination therapies is still limited, and the potential of producing viral escape mutants cannot be ruled out. An alternative, albeit less specific antiviral therapy is interferon (IFN) which, however, is only effective against a limited number of viral pathogens [15]. Moreover, because IFN treatment is prohibitively expensive and burdened with adverse side-effects, the therapy often results in low patient compliance [16,17]. These characteristics make IFN impractical for widespread use in clinical settings. In view of these shortcomings, there is a clearly a need to develop novel and cost-effective antiviral therapeutics, particularly those that harbor broad-spectrum bioactivities, which can be employed to control and limit the spread of viral infections when immunization and standard therapies are unavailable.

Glycosaminoglycans (GAGs) are negatively charged linear polysaccharides that are typically sulfated and include chondroitin sulfate (CS) and heparan sulfate (HS). They represent a repertoire of complex natural glycans that are localized within extracellular matrices and on cell surfaces, and exhibit heterogeneous structures that allow them to bind to a wide range of protein partners such as adhesion molecules, chemokines, cytokines, growth factors, and matrix proteins [18]. Thus, GAGs play important roles in many biologic processes, which have profound physiological consequences that include cell signaling, inflammation, angiogenesis, and coagulation [18,19]. Many viruses employ GAGs as primary entry factors that facilitate the infection of the host cell. These include DENV, HCMV, HCV, HIV, HSV, MV, RSV, and others [20-32]. Interactions of viral glycoproteins with GAGs are usually thought to increase the frequency of initial attachment of viral particles to the target cell surface. They, in turn, enable subsequent higher affinity binding with virus-specific entry receptors that promote virus entry. The importance of GAGs in facilitating viral infections has been demonstrated by using soluble heparin or GAG-deficient cell lines to block the entry of several viruses [20-31].

**Figure 1:**
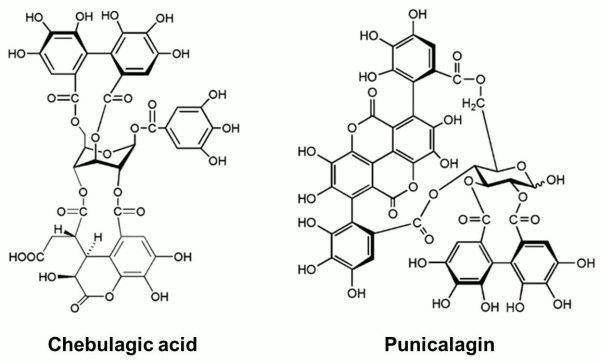
**Structures of chebulagic acid (CHLA) and punicalagin (PUG).** The chemical structures of the two hydrolyzable tannins under study, chebulagic acid (CHLA) and punicalagin (PUG), are presented.

In our previous study, we identified chebulagic acid (CHLA) and punicalagin (PUG) (Figure 1), two hydrolyzable tannins isolated from *Terminalia chebula* Retz., (*T. chebula*) as inhibitors of HSV type 1 (HSV-1) entry and spread [33]. We demonstrated that the two structurally-related compounds mediated their antiviral activities by targeting HSV-1 viral glycoproteins that interact with cell surface GAGs. Taking note of the fact that many viruses employ GAGs to initially bind to the host cell, and based on evidence that CHLA and PUG may act as GAG-competitors, we explored the antiviral-potential of these two tannins against a number of viruses known to interact with GAGs. Viral models included DENV, HCMV, HCV, MV, and RSV (Table 1). Many of the diseases associated with these viruses lack preventative vaccines and/or drug treatment options [1-4,13,34-36]. Indeed, both CHLA and PUG efficiently inhibited entry and spread of these viruses to varying degrees. We suggest that CHLA and PUG have potential as novel cost-effective and broad-spectrum antivirals for controlling emerging/recurring infections by viruses that engage host cell surface GAGs.

**Table 1:** Viruses used in this study and their requirement of cellular surface glycosaminoglycans for entry

**Virus**	**Family**	**Genome type**	**Envelope**	**Involvement of cellular surface glycosaminoglycans (GAGs) for entry**
HCMV	Herpesviridae	dsDNA	**+**	**+**
HCV	Flaviviridae	ssRNA (+)	**+**	**+**
DENV-2	Flaviviridae	ssRNA (+)	**+**	**+**
MV	Paramyxoviridae	ssRNA (-)	**+**	**+**
RSV	Paramyxoviridae	ssRNA (-)	**+**	**+**
VSV	Rhabdoviridae	ssRNA (-)	**+**	**?**
ADV-5	Adenoviridae	dsDNA	**-**	**+/-**

## Methods

### Cells, Viruses, and Reagents

Dulbecco’s modified Eagle’s medium (DMEM) and alpha minimal essential medium (AMEM) were purchased from GIBCO-Invitrogen (Carlsbad, CA, USA). Fetal bovine serum (FBS), penicillin G, streptomycin, and amphotericin B were purchased from Chemicon (Billerica, MA, USA). Heparin, dimethylsulfoxide (DMSO), and *in vitro* toxicology assay kit (XTT based) were purchased from Sigma (St. Louis, MO, USA).

Vero (African green monkey kidney cells, ATCC CCL-81), HEL (human embryonic lung fibroblast, ATCC CCL-137), and A549 (human lung carcinoma, ATCC CCL-185) cells were obtained from the American Type Culture Collection (ATCC; Rockville, MD, USA) and cultured in DMEM supplemented with 10% FBS, 200 U/ml penicillin G, 200 μg/ml streptomycin, and 0.5 μg/ml amphotericin B. Huh-7.5 (human hepatocarcinoma Huh-7 cell derivative; provided by Dr. Charles M. Rice, The Rockefeller University, New York, NY, USA) and HEp-2 (human epithelial cells derived from a larynx carcinoma; provided by R. Anderson) cells were cultured in the same medium condition as just described. CHO-SLAM or Chinese hamster ovary cells expressing human signaling lymphocyte activation molecule, the receptor for wild-type measles, were generated as previously reported and cultured in AMEM supplemented with 10% FBS and 800 μg/ml of G418 [37,38].

HCMV (AD169 strain; provided by Dr. Karen L. Mossman, McMaster University, Hamilton, ON, Canada), wild-type human adenovirus type-5 (ADV-5), and VSV-GFP (vesicular stomatitis virus with green fluorescent protein tag) have been described elsewhere and viral titers and antiviral assays were determined by standard plaque assay using methanol fixation followed by crystal violet (Sigma) [33,39,40]. Cell-culture derived HCV particles were produced by electroporation of Huh-7.5 cells using the Jc1FLAG2(p7-nsGluc2A) construct (genotype 2a; kindly provided by Dr. Charles M. Rice), which harbors a *Gaussia* luciferase reporter that allows detection of virus infectivity, as previously described [41]. HCV viral titer and antiviral assays were determined by immunofluorescence staining of TCID_50_ using anti-NS5A 9E10 antibody (gift from Dr. Charles M. Rice) and luciferase assays. DENV-2 (dengue virus type 2; strain 16681) and RSV (serogroup A, Long strain; ATCC VR-26) were propagated in Vero and HEp-2 cells, respectively [42,43]. Viral titers and antiviral assays for DENV-2 and RSV were determined by immunohistochemical staining plaque assay using anti-flavivirus group antibody (1:1,000; Millipore, Billerica, MA, USA), anti-RSV fusion protein antibody (1:5,000; Millipore), and goat anti-mouse IgG (H + L) alkaline phosphatase (AP) conjugate (Invitrogen; DENV-2, 1:5,000; RSV, 1:10,000), followed by development with Vector Black AP Substrate Kit (Vector Laboratories; Burlingame, C, USA) based on previously reported method [42]. MV-EGFP (recombinant Ichinose-B 323 wild-type measles virus isolate, IC323) expressing enhanced green fluorescent protein was originally obtained from Dr. Roberto Cattaneo (Mayo Clinic, Rochester, MN, USA) and propagated in marmoset B lymphoblastoid cells (B95a) [44]; viral titer and antiviral assays were determined by TCID_50_ on CHO-SLAM cells. The basal medium containing 2% FBS with antibiotics was used for all virus infection experiments. Virus concentrations are expressed as plaque forming units (PFU) per well or multiplicity of infection (MOI).

### Test compounds

CHLA and PUG (Figure 1) were isolated and purified as previously described, with their structures confirmed by high-performance liquid chromatographic method coupled with UV detection and electrospray ionization mass spectrometry (HPLC-UV/ESI-M), and their purities checked by HPLC with photodiode array detection (HPLC-PDA) [33]. Both compounds were dissolved in DMSO and the final concentration of DMSO was equal to/or below 1% for the experiments. Heparin served as control and was dissolved in sterile double-distilled water. For all assays, unless otherwise specified, test compound concentrations used were as follows based on antiviral dose response determined for each specific virus: HCMV (CHLA = 60 μM, PUG = 40 μM, Heparin = 30 μg/ml); HCV (CHLA = 50 μM, PUG = 50 μM, Heparin = 1000 μg/ml); DENV-2 (CHLA = 25 μM, PUG = 25 μM, Heparin = 200 μg/ml); MV (CHLA = 90 μM, PUG = 50 μM, Heparin = 10 μg/ml); RSV (CHLA = 1 μM, PUG = 2 μM, Heparin = 1 μg/ml).

### Cytotoxicity assay

Cells (1 × 10^4^ per well of 96-well plate) were treated with the test compounds for 3 days. Treatment effects on cell viability (%) and the 50% cytotoxic concentration (CC_50_) values of the test compounds were determined based on the XTT (2,3-*bis*[2-methoxy-4-nitro-5-sulfophenyl]-5-phenylamino)-carbonyl]-2*H*-tetrazolium hydroxide) assay as previously reported [33].

### Dose–response assay for measuring antiviral activities

**Figure 2:**
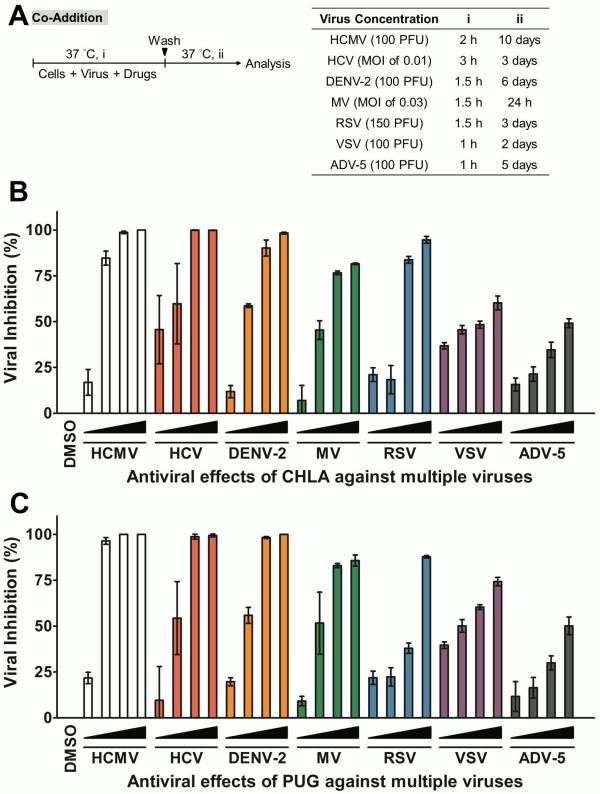
**Dose response of CHLA and PUG treatments against multiple viruses.** Host cells for each virus (HEL for HCMV; Huh-7.5 for HCV; Vero for DENV-2, CHO-SLAM for MV; HEp-2 for RSV, and A549 for VSV and ADV-5) were co-treated with viral inoculum and increasing concentrations of test compounds for 1 – 3 h before being washed, incubated, and analyzed for virus infection by plaque assays, EGFP expression analysis, or luciferase assay as described in Methods. **(A)** Schematic of the experiment (shown on the left) with the virus concentration (PFU/well or MOI), co-treatment time (i), and the subsequent viral incubation period (ii) indicated for each virus in the table on the right. **(B)** Antiviral effect of CHLA against multiple viruses. **(C)** Antiviral effect of PUG against multiple viruses. Results are plotted against values for the DMSO control treatment of virus infections and the data shown are means ± the standard errors of the mean (SEM) from three independent experiments. See text for details.

The respective cell lines and relative viral dose used, as well as the incubation periods for test compound treatment and for viral cytopathic effects to take place, are indicated in Table 2 and Figure 2A for each specific virus.

**Table 2:** **Cytotoxicity and antiviral activity of CHLA and PUG against different virus infections**^**a**^

**Virus**	**Cell type**	**Compounds**	**CC**_**50**_**(μM)**^**b**^	**Antiviral effect**	
				**EC**_**50**_**(μM)**^**c**^	**SI**^**d**^
HCMV	HEL	CHLA	306.32 ± 7.00	25.50 ± 1.51	12.01
		PUG	299.32 ± 9.14	16.76 ± 0.88	17.86
HCV	Huh-7.5	CHLA	237.61 ± 4.53	12.16 ± 2.56	19.54
		PUG	222.61 ± 3.41	16.72 ± 2.55	13.31
DENV-2	Vero	CHLA	159.63 ± 7.46	13.11 ± 0.72	12.18
		PUG	151.44 ± 9.31	7.86 ± 0.40	19.27
MV	CHO-SLAM	CHLA	351.83 ± 4.54	34.42 ± 4.35	10.22
		PUG	283.76 ± 11.54	25.49 ± 2.94	11.13
RSV	HEp-2	CHLA	244.17 ± 17.40	0.38 ± 0.05	642.55
		PUG	264.83 ± 23.72	0.54 ± 0.04	490.43
VSV	A549	CHLA	316.87 ± 9.01	61.28 ± 5.50	5.17
		PUG	318.84 ± 4.99	36.98 ± 4.59	8.62
ADV-5	A549	CHLA	316.87 ± 9.01	198.14 ± 14.07	1.60
		PUG	318.84 ± 4.99	196.67 ± 20.05	1.62

^a^ Values shown are means obtained from three independent experiments with each treatment performed in triplicate.

^b^ Cytotoxic effects were evaluated by XTT assay to determine the concentration of 50% cellular cytotoxicity (CC_50_) of the tested compounds.

^c^ Antiviral effects were evaluated by infection analysis to determine the effective concentration that achieved 50% inhibition (EC_50_) against the specific virus examined.

^d^ SI, selectivity index. SI = CC_50_/EC_50_.

For assessing the antiviral activities of the tannins on the panel of viruses, HEL (1 × 10^5^ cells/well), Vero (2 × 10^5^ cells/well), HEp-2 (1.5 × 10^5^ cells/well), and A549 (2 – 3 × 10^5^ cells/well) cells were seeded in 12-well plates and co-treated with the respective viral inoculum (Figure 2A) and increasing concentration of test compounds for 1 – 2 h. The inoculum and drug mixtures were removed from the wells that were subsequently washed with PBS twice and then overlaid with 2% FBS medium containing either methylcellulose (Sigma; HCMV: 0.6%; DENV-2: 0.75%; RSV and VSV: 1%) or SeaPlaque agarose (Lonza, Basel, Switzerland; ADV-5: 1%). After further incubation for 24 h – 10 days depending on the specific virus, wells containing ADV-5, HCMV, and VSV infections were analyzed by standard plaque assays, and wells containing DENV-2 and RSV infections were analyzed by immunohistochemical staining as described above. Viral infection (%) and the 50% effective concentration (EC_50_) of test compounds against different viral infections were calculated as previously described [33].

For evaluating the antiviral activities of the tannins on MV-EGFP infection, CHO-SLAM cells (2 × 10^4^ cells/well) were seeded in 96-well plates and viral inoculum and increasing concentration of the test compounds were co-added onto the cell monolayer for 1.5 h. The inoculum and drug mixtures were then removed and the wells were washed with PBS twice before overlay with AMEM containing 2% FBS. After further incubation for 24 h, the plates were then scanned by the Typhoon 9410 variable mode imager (Amersham Biosciences; Baie d’Urfe, Quebec, Canada) and the EGFP expression was analyzed by ImageQuant TL software (Amersham Biosciences). Viral inhibition (%) and the EC_50_ for each compound based on viral EGFP expression were determined as previously reported [33].

For analyzing antiviral activities of the tannins on HCV infection, Huh-7.5 cells (1 × 10^4^ cells/well) were seeded in 96-well plates and the cell monolayer was co-challenged with the viral inoculum and increasing concentration of the test compounds for 3 h. The inoculum and drug mixtures were removed from the wells, followed by washing with PBS twice and overlaying with DMEM containing 2% FBS. After further incubation for 72 h, the supernatant was collected and then assayed for luciferase activity using the BioLux™ Gaussia Luciferase Assay Kit (New England Biolabs; Pickering, ON, Canada) and a luminometer (Promega; Madison, WI, USA). HCV infectivity was expressed as log_10_ of relative light units (RLU) for determining viral inhibition (%) and the EC_50_ of the drugs against HCV infection was calculated using GraphPad Prism 5 software (San Diego, CA, USA).

All values were plotted against the DMSO control treatment of virus infection.

### Viral inactivation assays

**Figure 3:**
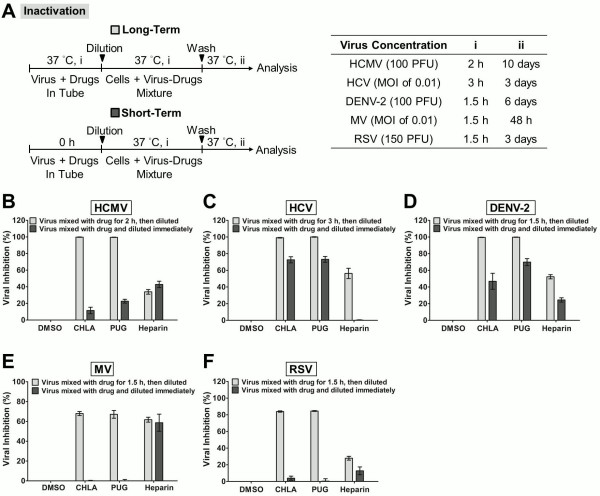
**Inactivation of viral infections by CHLA and PUG.** Different viruses were treated with the test compounds for a long period (incubated for 1.5 – 3 h before titration; light gray bars) or short period (immediately diluted; dark gray bars) at 37°C before diluting it 50 – 100 fold to sub-therapeutic concentrations and subsequent analysis of infection on the respective host cells. **(A)** Schematics of the experiment (shown on the left) with the final virus concentration (PFU/well or MOI), long-term virus-drug incubation period (i), and the subsequent incubation time (ii) indicated for each virus in the table on the right. Analyses for **(B)** HCMV, **(C)** HCV, **(D)** DENV-2, **(E)** MV, and **(F)** RSV are indicated in each additional panel. Results are plotted against the DMSO negative control treatment for virus infection and the data shown are the means ± SEM from three independent experiments. See text for details.

Viral inactivation assays were performed as previously described [33] and the incubation periods and viral dose used are listed in Figure 3A. Different viruses were mixed with the test compounds and incubated at 37°C (Figure 3A, long-term). The drug-virus mixtures were subsequently diluted (50 – 100 fold) to “sub-therapeutic” (ineffective) concentrations with low serum medium and then inoculated on to the respective host cells seeded in multiwell plates. The dilution to sub-therapeutic concentration prevents effective interaction between the drugs and the host cell surface. For comparison, viruses were also mixed with test compounds and immediately diluted (no incubation period) to sub-therapeutic concentration prior to infection (Figure 3A, short-term). Following incubation for viral absorption, the diluted inocula were removed and the wells were washed with PBS twice before applying the overlay medium. The plates were further incubated before being subjected to assessment by plaque assays, EGFP expression analysis, or luciferase assay as described above.

### Viral attachment assays

Analyses of drug effect on viral attachment were performed based on host cell infection (method 1) or virus-specific cellular enzyme-linked immunosorbent assay (ELISA; method 2) as previously described [33]. Experiments were all carried out at 4°C which allows for virus binding but precludes entry which occurs most efficiently at 37°C.

**Figure 4:**
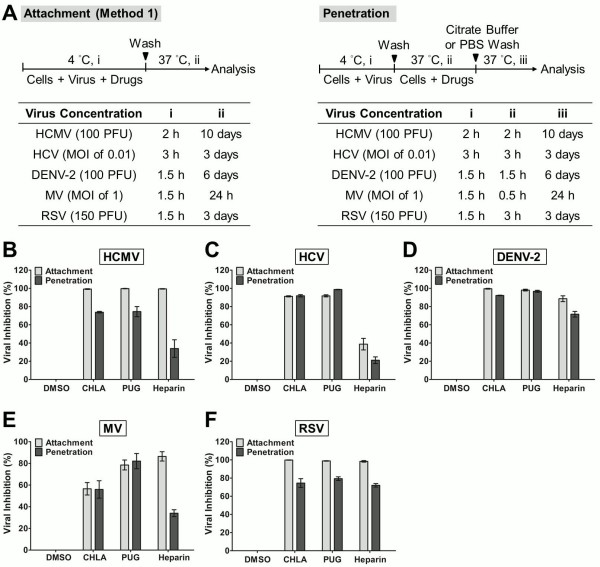
**Evaluation of antiviral activities of CHLA and PUG that affect virus attachment and penetration. (A)** Schematics of the experiments with the virus concentration (PFU/well or MOI) and the time of addition and treatment with tannins (i, ii, iii) for each virus in the associated tables. In virus attachment analysis by Method 1 (light gray bars), monolayers of different cell types were pre-chilled at 4°C for 1 h, and then co-treated with the respective viruses and test compounds at 4°C (1.5 – 3 h; i) before washing off the inoculates and test compounds for subsequent incubation (37°C; ii) and examination of virus infection. In virus penetration analysis (dark gray bars), seeded cell monolayers were pre-chilled at 4°C for 1 h and then challenged with the respective viruses at 4°C for 1.5 – 3 h (i). Cells were then washed and treated with the test compounds for an additional incubation period (ii) during which the temperature was shifted to 37°C to facilitate viral penetration. At the end of the incubation, extracellular viruses were removed by either citrate buffer (pH 3.0) or PBS washes and the cells were further incubated (iii) for analysis of virus infection. Results for **(B)** HCMV, **(C)** HCV, **(D)** DENV-2, **(E)** MV, and **(F)** RSV are indicated in each additional panel. Data are plotted against the DMSO negative control treatment of virus infection and are presented as means ± SEM from three independent experiments. See text for details.

In method 1 (Figure 4A), different cell types were pre-chilled at 4°C for 1 h and then co-treated with dose of respective viruses and test compounds at 4°C for the indicated times. The inocula and drugs were removed and the cell monolayers were washed with ice-cold PBS twice before applying the overlay medium. After further incubation at 37°C, plaque assays, EGFP expression analysis, or luciferase assay were performed as described above to assess host cell infection.

**Figure 5:**
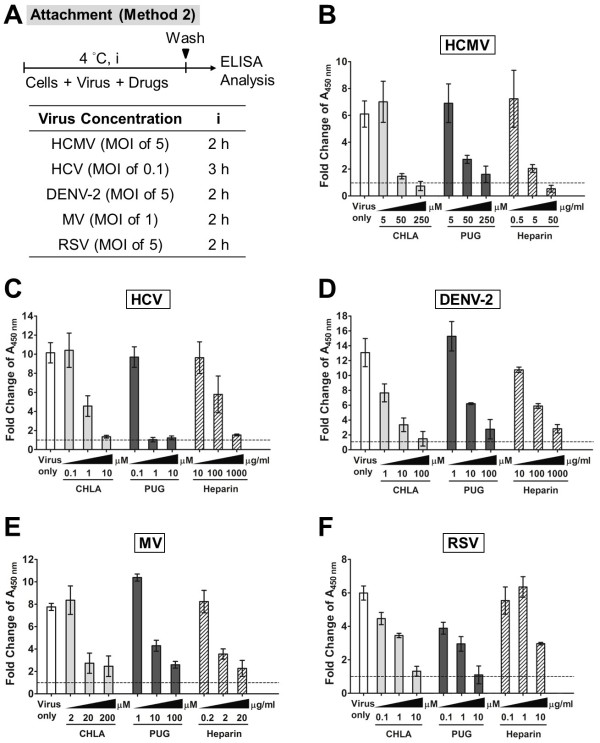
**Effects of CHLA and PUG against virus binding analyzed by ELISA.** Different cell monolayers were pre-chilled at 4°C for 1 h and then inoculated with the respective viruses in the presence or absence of various concentrations of test compounds at 4°C for an additional 2 h. Following the virus binding period, the cell monolayers were washed to remove unadsorbed virus, subsequently fixed with 4% PFA, and then blocked with 5% BSA. ELISA was performed with virus-specific antibodies and HRP-conjugated IgG, followed by development with a TMB substrate kit. The absorbance was immediately determined at 450 nm and values are expressed as the fold change of absorbance relative to the mock infection control (cells + DMSO), which is indicated by the dashed line. **(A)** Schematic of the experiment with the virus concentration (MOI) and test compound treatment time (i) indicated for each virus in the associated table. Analyses for **(B)** HCMV, **(C)** HCV, **(D)** DENV-2, **(E)** MV, and **(F)** RSV are indicated in each additional panel. Results shown are means ± SEM from three independent experiments. See text for details.

In method 2 (Figure 5A), different cell types (2 × 10^4^ cells/well) were seeded in 96-well plates and grown overnight. The cell monolayers were pre-chilled at 4°C for 1 h and then co-treated with the respective viruses (HCMV, MOI = 5; HCV, MOI = 0.1; DENV-2, MOI = 5; MV, MOI = 1; RSV, MOI = 5) and various concentrations of test compounds at 4°C for an additional 2 h. Following the virus binding period, the inocula and drugs were removed and the cell monolayers were washed with ice-cold PBS before fixation with pre-chilled 4% paraformaldehyde (PFA) in PBS for 1 h on ice. At that point, the wells were blocked with 5% bovine serum albumin (BSA) at 4°C overnight to prevent any non-specific binding. Bound viruses on the cellular surfaces were then detected by ELISA assay whereby wells were incubated with the following respective mouse monoclonal primary antibodies (diluted in PBS containing 5% BSA) at 37°C for 1 h before washing with PBST (0.1% Tween 20 in PBS) three times: anti-HCMV gB antibody (1:10,000; Thermo Pierce, Rockford, IL, USA), anti-HCV E2 antibody (1:20,000; AUSTRAL Biologicals, San Ramon, CA, USA), anti-flavivirus group antibody (1:5,000) for DENV-2, anti-measles hemagglutinin antibody (1:5,000; Millipore), and anti-RSV fusion protein antibody (1:15,000). Samples were then subjected to incubation at 37°C for 1 h with goat anti-mouse IgG conjugated with horseradish peroxidase (HRP; Invitrogen), diluted at 1:20,000 (HCMV, DENV-2, MV-EGFP), 1:36,000 (HCV), or 1:30,000 (RSV) in PBS containing 5% BSA. The wells were afterwards washed with PBST three times and developed with a TMB (3,3′,5,5′-tetramethylbenzidine) Two-component Microwell Peroxidase Substrate Kit (KPL, Gaithersburg, MD) at room temperature for 20 min before stopping the reaction with 1 M phosphoric acid (H_3_PO_4_). The plates were measured with an ELx800 Microplate reader (Instrument, Inc.; Winooski, VT, USA) at 450 nm.

### Viral penetration assay

The viral penetration assay was performed as previously reported [33] and the incubation periods and viral dose used are indicated in Figure 4A. Monolayers of different cell types were pre-chilled at 4°C for 1 h and then infected for the indicated times with the respective viruses at 4°C to allow virus binding but not entry. The inocula were removed and the wells were washed with ice-cold PBS twice before treating with the test compounds for the indicated times at 37°C. This shift to 37°C facilitates viral penetration and therefore allows assessment of drug effect on viral internalization. The drugs were afterwards removed and non-internalized extracellular viruses were detached by either citrate buffer (50 mM Sodium Citrate, 4 mM KCl, pH 3.0) or PBS washes. The wells were then further washed with PBS twice prior to covering the cell monolayers with overlay medium. After additional incubation at 37°C, plaque assays, EGFP expression analysis, or luciferase assay were performed as described above.

### Analysis of drug effects post viral entry

**Figure 6:**
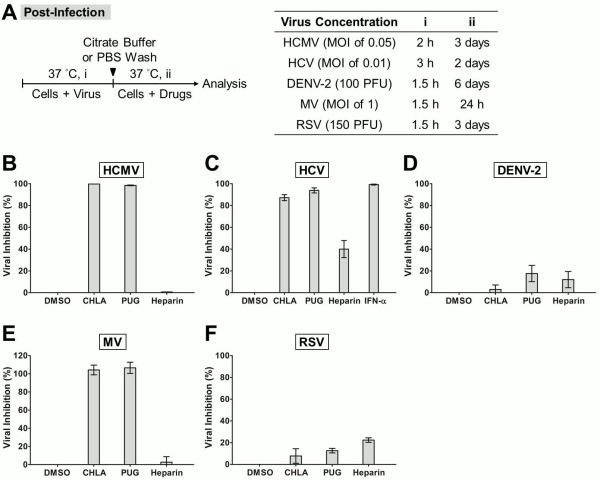
**Post-infection analysis of antiviral effects due to CHLA and PUG.** Cell monolayers were inoculated with the respective viruses at 37°C to allow viral entry, then washed by citrate buffer or PBS to remove extracellular viruses, and subsequently incubated in the presence or absence of the test compounds for infection analysis. **(A)** Schematic of the experiment (left) with the virus concentration (PFU/well or MOI), virus infection time (i), and test compound treatment period post-infection (ii) indicated for each virus in the table shown on the right. Results for **(B)** HCMV, **(C)** HCV, **(D)** DENV-2, **(E)** MV, and **(F)** RSV are indicated in each additional panel. IFN-α treatment was included as control for HCV infection. Data shown are means ± SEM from three independent experiments. See text for details.

For examining drug effects post viral entry, cell monolayers were infected with respective viruses at 37°C with the viral dose and incubation times as specified in Figure 6A. Following the absorption period, the inocula were removed and extracellular viruses were detached by citrate buffer or PBS washes as just described before treating with the test compounds mixed in the overlay medium at 37°C for the indicated times. Plaque assay, EGFP expression assessment, or luciferase assay were performed as described above for analysis. For HCMV, the infection was titered by standard plaque assay on newly seeded HEL cells. Alpha interferon (IFN-α) from human leukocytes (1,000 U/ml; Sigma) was included as control for HCV.

### Viral cell-to-cell spread assay

**Figure 7:**
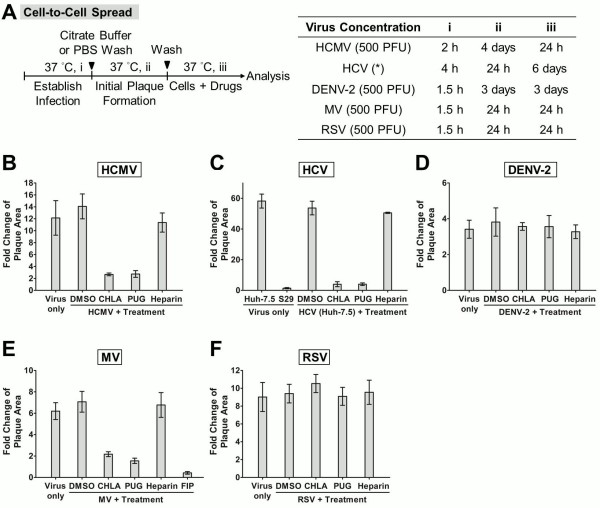
**Examination of CHLA and PUG treatment on virus cell-to-cell spread. (A)** Schematic of the experiment (left) with the virus concentration (PFU/well) and step-wise incubation periods (i, ii, iii) indicated for each virus in the table on the right. Virus infections were established (i) in the different cell types by direct inoculation (HCMV, DENV-2, MV, and RSV) or electroporation of viral RNA (HCV; *), and the cell monolayers were washed with citrate buffer or PBS before being covered with an overlay medium that prevents secondary infection. Initial virus plaques were allowed to form in the subsequent infections (ii), and then the test compounds were added to the overlay medium for an additional time of incubation (iii) before analysis of viral plaque size by immune fluorescence microscopy. Five random virus-positive plaques at the endpoint of the experiment were evaluated for each treatment group of viruses, and the data was plotted as “fold change of plaque area” against the size of the initial viral plaques formed prior to test compound treatment. Analyses for **(B)** HCMV, **(C)** HCV, **(D)** DENV-2, **(E)** MV, and **(F)** RSV are indicated in each additional panel. The S29 cell line and the FIP inhibitor were included as controls for HCV and MV, respectively. Results shown are means ± SEM from three independent experiments and representative micrographs of the evaluated plaques are provided in Additional file 1 Figure S1, Additional file 2 Figure S2, Additional file 3 Figure S3, Additional file 4 Figure S4 and Additional file 5 Figure S5. See text for details.

Viral cell-to-cell spread assay was performed as previously described [33,45] with some modifications and the viral dose and incubation periods are indicated in Figure 7A. Briefly, different cell types were infected with the respective viruses and extracellular viruses were removed by citrate buffer or PBS washes as specified earlier. The wells were then covered with overlay medium containing either methylcellulose (DENV-2: 0.75%; RSV: 1%), SeaPlaque agarose (Lonza; MV: 1%), or in the case of HCMV with 0.1% of neutralizing Gamunex antibodies (purified clinical human IgGs; provided by Dr. Andrew C. Issekutz, Dalhousie University, Halifax, NS, Canada) [33]. The overlay medium helps limit viral secondary infection, thus allowing monitoring of cell-to-cell spread of virus in the presence or absence of the drugs. The plates were incubated until initial plaque formation, to which the test compounds were then added into the overlay medium and monitored in subsequent incubation for analysis of viral plaque size by immunofluorescence assay. The fusion inhibitory peptide (FIP, Z-D-Phe-L-Phe-Gly-OH, 200 μM; Sigma) also served as control for MV [46].

The examination of HCV spread is based on previously described protocol with some modifications [47]. Huh-7.5 cells were electroporated with HCV Jc1FLAG2(p7-nsGluc2A) RNA (10 μg) as described above to establish random productive infections in the cell population, and then mixed with naïve cells at a ratio of 1:1 before seeding in 12-well plates. Assembled HCV particles (within 24 – 48 h post-transfection) would transmit to neighboring cells that do not harbor viral RNA during viral spread and form localized foci in ensuing incubation period [48]. Medium was changed 24 h post-electroporation with an overlay medium containing the test drugs or control and 0.5% methylcellulose, and the plates were further incubated for 5 days before analysis of HCV-positive foci through immunostaining. The S29 cell line (provided by Dr. Rodney S. Russell, Memorial University of Newfoundland, St. John’s, NL, Canada), which is a Huh-7 derivative deficient in the HCV receptor CD81, does not allow cell-to-cell transmission of HCV infection and was included as control [49].

For immunofluorescence analysis of viral plaque size due to spread, the overlay media were removed and the wells were fixed with ice-cold methanol before blocking with 3% BSA. Samples were then treated at 37°C for 1 h with the respective mouse monoclonal primary antibodies diluted in PBS containing 3% BSA: anti-HCMV gB antibody (1:1,000), anti-NS5A 9E10 antibody for HCV (1:25,000), anti-flavivirus group antibody (1:400) for DENV-2, and anti-RSV fusion protein antibody (1:1,000). After incubation, the wells were washed with PBS three times before applying Alexa Fluor 488 goat anti-mouse IgG (H + L) antibody (Invitrogen), diluted at 1:1,000 (HCMV and RSV) or 1:400 (DENV-2 and HCV) in PBS containing 3% BSA. Following incubation at 37°C for 1 h, the samples were washed with PBS three times prior to visualization by fluorescence microscopy. The fluorescence expression of MV-EGFP could be readily detected without addition of antibodies. Photomicrographs were taken at × 100 magnification (Leica Microsystems; Wetzlar, Germany) and viral plaque sizes were then analyzed with MetaMorph software (Molecular Devices; Sunnyvale, CA, USA). In the case of HCV, cellular nuclei were stained with Hoechst dye (Sigma) prior to visualization and the number of cells in the virus-positive foci was determined. For all virus tested, a total of five random virus-positive plaques were evaluated for each treatment group per independent experiment. Comparison was made between viral plaques stained prior to drug addition and those at the endpoint of the experiment, and the data were plotted as “fold change of plaque area”.

## Results

### Broad-spectrum antiviral effects of CHLA and PUG

CHLA and PUG were evaluated for their antiviral effects against a panel of enveloped viruses whose entry involves cellular surface GAGs (Table 1). Vesicular stomatitis virus (VSV) and adenovirus type 5 (ADV-5) were included for comparison. The 50% indices of cytotoxicity (CC_50_) and effective antiviral concentrations (EC_50_), as well as the selective index (SI = CC_50_/EC_50_), were determined for each virus infection host cell system and are listed in Table 2. As shown in Figure 2, CHLA and PUG displayed broad-spectrum antiviral effects in a dose-dependent manner. Both compounds exhibited significant inhibitory effect on enveloped viruses known to engage GAGs for infection, including HCMV, HCV, DENV-2, MV, and RSV, with their EC_50_ < 35 μM and SI > 10 (Table 2). Both tannins were especially effective against RSV with their EC_50_ values being < 1 μM. The two compounds, however, displayed only limited efficacy (SI < 10) against infections by VSV and ADV-5. This is consistent with the fact that these viruses have previously been shown not to require GAGs for entry. VSV can infect GAG-deficient cells [22,23,50], whereas HS-mediated entry is only important for ADV-5 in the absence of its primary receptor CAR (*c*oxsackievirus and *a*denovirus *r*eceptor) and can only be inhibited to a maximum of 50% by soluble heparin [51,52]. For the remainder of the studies, we focused on the effects of the tannins against HCMV, HCV, DENV-2, MV, and RSV.

### Free virus particles are inactivated by CHLA and PUG

CHLA and PUG were previously observed to inactivate HSV-1 particles and prevent their interaction with the host cell surface [33]. We examined whether the tannins could also inactivate the different enveloped viruses and prevent subsequent infection. These natural products were pre-incubated with the viruses and then diluted to sub-therapeutic concentrations prior to infecting the respective host cell. Results indicated that both CHLA and PUG were able to interact with HCMV, HCV, DENV-2, MV, and RSV virions. Their effects were irreversible and abrogated subsequent infections (Figure 3). A 60 – 80% block against the paramyxoviruses MV and RSV was observed, whereas near 100% inhibition was achieved against HCMV, HCV, and DENV-2. The data suggest that CHLA and PUG can directly inactivate these free virus particles and neutralize their infectivity.

### CHLA and PUG inhibit virus entry-related steps

In further characterizing the antiviral mechanism(s) involved, we explored the effect of CHLA and PUG against HCMV, HCV, DENV-2, MV, and RSV attachment to the host cell surface and upon subsequent membrane fusion. The temperature change between 4°C (permitting virus binding but not entry) and 37°C (facilitating virus entry/penetration) allows examination of the drug effect on each specific event [53]. Both tannin compounds effectively prevented attachment of the investigated viruses as shown by readouts of inhibition of infection (method 1; Figure 4) and by ELISA-based binding assays using virus-specific antibodies to detect bound virus on the cell monolayer (method 2; Figure 5). The inhibition of virus attachment by CHLA and PUG were similar against HCMV, HCV, DENV-2, and RSV, and ranged from 90 – 100% (Figure 4). Against MV, PUG appeared to be more effective than CHLA, and inhibition of entry varied between 50 – 80%. The compounds’ ability to abolish binding of the above viruses was confirmed by the decrease of virions detected on cell surfaces. This occurred in a dose-dependent manner with increasing concentrations of the tannins (Figure 5).

To see whether the CHLA and PUG retained their activity during the virus penetration phase, the test viruses were allowed to bind to the cell surface at 4°C and then allowed to penetrate the target cell membrane by a temperature shift to 37°C in the presence or absence of the tannins. CHLA and PUG were again observed to impair virus entry by these viruses, resulting in 50 – 90% protection of the host cell from infection from the virus being examined (Figure 4). Heparin exhibited similar inhibitory effects as the tannins against attachment of the test viruses, but was less potent in inhibiting cell penetration by HCMV, HCV, and MV (< 40% inhibition on average). Therefore, CHLA and PUG are able to abrogate host cell binding and penetration by HCMV, HCV, DENV-2, MV, and RSV during the cell entry process.

### Control of virus spread post-infection by CHLA and PUG

We next determined the antiviral activity of the two hydrolyzable tannins in controlling spread of established infections. Target cell monolayers were infected with the respective test virus, and then incubated with or without the compounds. As shown in Figure 6, both CHLA and PUG effectively inhibited HCMV, HCV, and MV infections (80 – 100% protection), but were ineffective against the growth of DENV-2 and RSV (< 25%). To further validate the tannins’ effect on virus cell-to-cell transmission, we examined the effects of the drugs on viral plaque size. The change in the area of the plaques was measured using either viral immunofluorescence or EGFP-tagged reporter viruses. Neutralizing antibodies, methylcellulose or agarose were included in the overlay medium to prevent secondary infection of uninfected cells throughout the monolayer, ensuring that viral spread occurs via intercellular junctions between neighboring infected and virus-free populations. The data indicated that viral plaques from HCMV, HCV, and MV infections were restricted by CHLA and PUG to near initial size, whereas plaques due to DENV-2 and RSV infections were unaffected and expanded further (Figure 7 and Additional file 1: Figure S1, Additional file 2: Figure S2, Additional file 3: Figure S3, Additional file 4: Figure S4 and Additional file 5: Figure S5). These results are in agreement with the data obtained following post-entry drug treatment in Figure 6, where HCMV, HCV, and MV, but not DENV-2 and RSV, were shown to be sensitive to the tannins’ antiviral effects. Thus, it appears that the two tannins are effective in limiting post-infection spread of HCMV, HCV, and MV, but are inefficient in preventing cell-to-cell transmission of DENV-2 and RSV. Heparin, on the other hand, displayed limited effect against the spread of the viruses post-entry (Figures 6 and 7). The window of antiviral activity from CHLA, PUG, and heparin at different stages of viral entry and spread are summarized in Table 3.

**Table 3:** Window of antiviral effects from CHLA and PUG against different viruses

**Virus**	**Treatments**	**Window of antiviral effects**^**a**^					
		**Free particle**	**Attachment**		**Penetration**	**Post-infection**	**Cell-to-cell spread**
			**Method 1**	**Method 2**^**b**^			
HCMV	CHLA	+++	++	+++	++	+++	+++
	PUG	+++	++	++	++	+++	+++
	Heparin	+	++	+++	+	-	-
HCV	CHLA	+++	+++	+++	+++	+++	+++
	PUG	+++	+++	+++	+++	+++	+++
	Heparin	++	+	+++	-	+	-
DENV-2	CHLA	+++	+++	+++	+++	-	-
	PUG	+++	+++	+++	+++	-	-
	Heparin	++	+++	+++	++	-	-
MV	CHLA	++	++	++	++	+++	+++
	PUG	++	+++	++	+++	+++	+++
	Heparin	++	+++	++	+	-	-
RSV	CHLA	+++	+++	+++	++	-	-
	PUG	+++	+++	+++	+++	-	-
	Heparin	+	+++	++	++	-	-

^a^ Inhibitory effect (%) relative to DMSO control: +++ > 75%; ++ 50–75%; + 25–50%; - < 25%. Heparin was included for comparison.

^b^ Results from the highest inhibitory concentration of the compounds were used.

## Discussion

The inhibition of virus-host cell entry is an effective antiviral control strategy. Based on the way a virus infects a host cell through interactions between viral glycoproteins and cellular membrane molecules, countermeasures against this process have been developed. For example, protective antibodies elicited by vaccines bind to viral particles and prevent infection [54]. Another strategy consists of using monoclonal antibodies or small molecules to bind host cell receptors and block virus interactions. Examples include an antibody directed against the HCV receptor claudin 1, and another is the antagonist maraviroc, which interacts with the HIV coreceptor CCR5 [14,55]. Another HIV inhibitor called enfuvirtide blocks gp41-mediated membrane fusion during virus entry. Amantidine blocks influenza M2 ion channel activity during entry and viral assembly [14,56]. On the other hand, non-specific approaches directed against the virus can influence membrane fluidity (lipid bilayer intercalator LJ001), membrane fusion (rigid amphipathic fusion inhibitors, RAFIs) [57,58], or neutralize surface charge (cationic amphipathic sterol, squalamine) [59]. These are effective against a wide range of enveloped viruses. Similarly, we recently considered GAG receptors as targets for potential antiviral therapy. Two natural molecules of the hydrolyzable tannin class, CHLA and PUG, possess GAG-competing properties [33]. In this study, both compounds displayed significant *in vitro* antiviral activity against a variety of viruses, suggesting that blocking interaction with GAGs is a feasible way to prevent infection by some viruses. Our finding adds to the list of molecular strategies that are being developed to prevent and limit viral infections.

We previously showed that CHLA and PUG exerted their antiviral effects against HSV-1 by binding viral glycoproteins that interact with cell surface GAGs [33]. In the current study, these compounds were demonstrated to be effective against infection by other viruses, including HCMV, HCV, DENV-2, MV, and RSV, whose entry is known to be sensitive to neutralization by heparin (Table 3). Similar to HSV-1 [33], the tannins are hypothesized to bind to viral glycoproteins on these viruses and the cell surfaces of infected cells, blocking virus attachment, entry, and cell to cell spread. The two tannins may target more than one step of infection, including attachment, membrane fusion, and cell-to-cell fusion. Many viral glycoproteins have multiple roles including binding to host cell surface GAGs, interaction with higher affinity receptors, and mediating membrane fusion [25,33,60-64]. Since CHLA and PUG could not block the spread of DENV-2 and RSV, this might reflect situations where the inhibitors interact with specific sites on the viral glycoproteins involved with attachment, membrane fusion, or cell spread, but not all these functions. Conformational changes in the viral glycoproteins could result from binding to the tannins and interactions with the cellular microenvironment may vary for the different viruses. For example, heparin was observed to be relatively ineffective against post-entry spread for all viruses examined. This could be due to the fact that the molecular size of heparin limits its accessibility to viral glycoproteins in the intercellular junctions [33]. In addition, the tannins displayed differential efficacies against the viruses examined (Table 2). It is interesting that CHLA and PUG appeared to be particularly selective against RSV, which could be due to higher affinity of the compounds against the RSV glycoproteins. Detailed structure-activity relationship (SAR) studies coupled with the analysis of individual viral glycoproteins would be necessary to clarify these issues. In addition, the use of genetically altered virus lacking certain glycoproteins, for example the DeltaG RSV with deleted glycoprotein G [31], could further help clarify the tannins’ antiviral mechanism.

Although vaccines represent the preferred method for protection against viruses, they have limited use against individuals who are already infected with a virus. Vaccines are also associated with problems of supply, cost of development, coverage and deployment, and efficacy against newly emerging and rapidly mutating viruses [65]. While some antiviral therapies have proven successful, treatment of many pathogenic viral infections have yet to be developed or approved. These include several of the infectious agents investigated in this study. The clinical value of current antiviral drugs is also frequently compromised by development of drug resistant variants causing recurrence of viral infections. Broad-spectrum antivirals may offer some relief in the treatment of these infections. Although many viruses use GAGs to initiate infection, therapies exploiting this interaction have yet to be developed. Heparin, which is also a type of GAG, is known to block the interaction of viral glycoproteins with GAGs in cell culture studies. However, it is not clinically useful *in vivo* for frequent/long-term administration due to side effects related to its anticoagulation activity [66]. Conversely, while the CHLA and PUG are structurally different from heparin, they also target the GAG-interacting properties of viruses and possess a much higher potency. *In vivo* toxicological and metabolic studies of these tannins have been explored with both showing minimal toxicity [67,68]. Furthermore, the two compounds could be mass-produced by chemical synthesis or extracted from *T. chebula*, which is widespread throughout Southeast Asia, making them attractive, cost-effective drug candidates [69-72]. Therefore, development of broad-spectrum antivirals using CHLA and PUG or their structure as lead compounds could be useful. They could help control viruses such as HCV, DENV, MV, and RSV, especially in epidemic areas and resource-poor countries where active vaccine or commercial programs are unavailable. Potential applications include formulations of the tannins as topical creams, gels, aerosol inhalers, or incorporating these compounds in materials, such as wipes, surgical masks, and protective gloves.

## Conclusions

In conclusion, we have demonstrated that CHLA and PUG have the ability to function as broad-spectrum antivirals *in vitro*. They effectively prevented infections by viruses utilizing GAG-assisted entry, and included HCMV, HCV, DENV, MV, and RSV. These natural molecules could serve as new therapeutic agents and help limit infections by viruses for which vaccines or FDA-licensed drugs do not yet exist. Future clinical applications and studies investigating their efficacy *in vivo* against specific viruses should be explored.

## Competing interests

The authors declare that they have no competing interests.

## Authors’ contributions

Conceived and designed the experiments: LTL. Performed the experiments: LTL TYC. Analyzed the data: LTL CCL CDR. Contributed reagents/materials/technical support: LTL TYC SCL CYC TCL GHW RA CCL CDR. Wrote and edited the paper: LTL CCL CDR. All authors read and approved the final manuscript.

## Supplementary Material

Additional file 1: Figure S1 Examination of CHLA and PUG treatment on HCMV cell-to-cell spread. HEL cell monolayers were inoculated and infected with HCMV for 2 h, washed with PBS to remove excess surface bound virus, and covered with an overlay medium to prevent secondary infection. Initial virus plaques were allowed to form in the subsequent infections and CHLA, PUG, Heparin, DMSO control were added to the overlay medium for an additional incubation time before analysis of viral plaque size by immune fluorescence microscopy at 5 days post-infection as described in Methods. Representative virus plaques/foci are shown after three independent experiments were performed. Scale bar indicates 100 μm. Click here for file

Additional file 2: Figure S2 Examination of CHLA and PUG treatment on HCV cell-to-cell spread. Huh-7.5 cells were electroporated with full-length HCV replicon RNA and covered with an overlay medium to prevent secondary infection. Initial virus plaques were allowed to form in the subsequent infections and CHLA, PUG, Heparin, and DMSO control were added to the overlay medium for an additional incubation time before analysis of viral plaque size by immune fluorescence microscopy at 7 days post-electroporation as described in Methods. Representative virus plaques/foci are shown after three independent experiments were performed. Scale bar indicates 100 μm. Click here for file

Additional file 3: Figure S3 Examination of CHLA and PUG treatment on DENV-2 cell-to-cell spread. Vero cells were inoculated and infected with DENV-2 for 1.5 h, washed with citrate buffer to remove excess surface bound virus, and covered with an overlay medium to prevent secondary infection. Initial virus plaques were allowed to form in the subsequent infections and CHLA, PUG, Heparin, and DMSO control were added to the overlay medium for an additional incubation time before analysis of viral plaque size by immune fluorescence microscopy at 6 days post-infection as described in Methods. Representative virus plaques/foci are shown after three independent experiments were performed. Scale bar indicates 100 μm. Click here for file

Additional file 4: Figure S4 Examination of CHLA and PUG treatment on MV-EGFP cell-to-cell spread. CHO-SLAM cells were inoculated and infected with MV-EGFP for 1.5 h, washed with citrate buffer to remove excess surface bound virus, and covered with an overlay medium to prevent secondary infection. Initial virus plaques were allowed to form in the subsequent infections and CHLA, PUG, Heparin, FIP, and DMSO control were added to the overlay medium for an additional incubation time before analysis of viral plaque size by EGFP fluorescence microscopy at 48 h post-infection as described in Methods. Representative virus plaques/foci are shown after three independent experiments were performed. Scale bar indicates 100 μm. Click here for file

Additional file 5: Figure S5 Examination of CHLA and PUG treatment on RSV cell-to-cell spread. HEp-2 cells were inoculated and infected with RSV for 1.5 h, washed with citrate buffer to remove excess surface bound virus, and covered with an overlay medium to prevent secondary infection. Initial virus plaques were allowed to form in the subsequent infections and CHLA, PUG, Heparin, and DMSO control were added to the overlay medium for an additional incubation time before analysis of viral plaque size by immune fluorescence microscopy at 48 h post-infection as described in Methods. Representative virus plaques/foci are shown after three independent experiments were performed. Scale bar indicates 100 μm. Click here for file
