# Supplementary figures and images for: Broad-spectrum antiviral activity of chebulagic acid and punicalagin against viruses that use glycosaminoglycans for entry
Source: BMC Microbiol. 2013 Aug 7;13:187. doi: 10.1186/1471-2180-13-187 (PMC3750913; doi:10.1186/1471-2180-13-187)

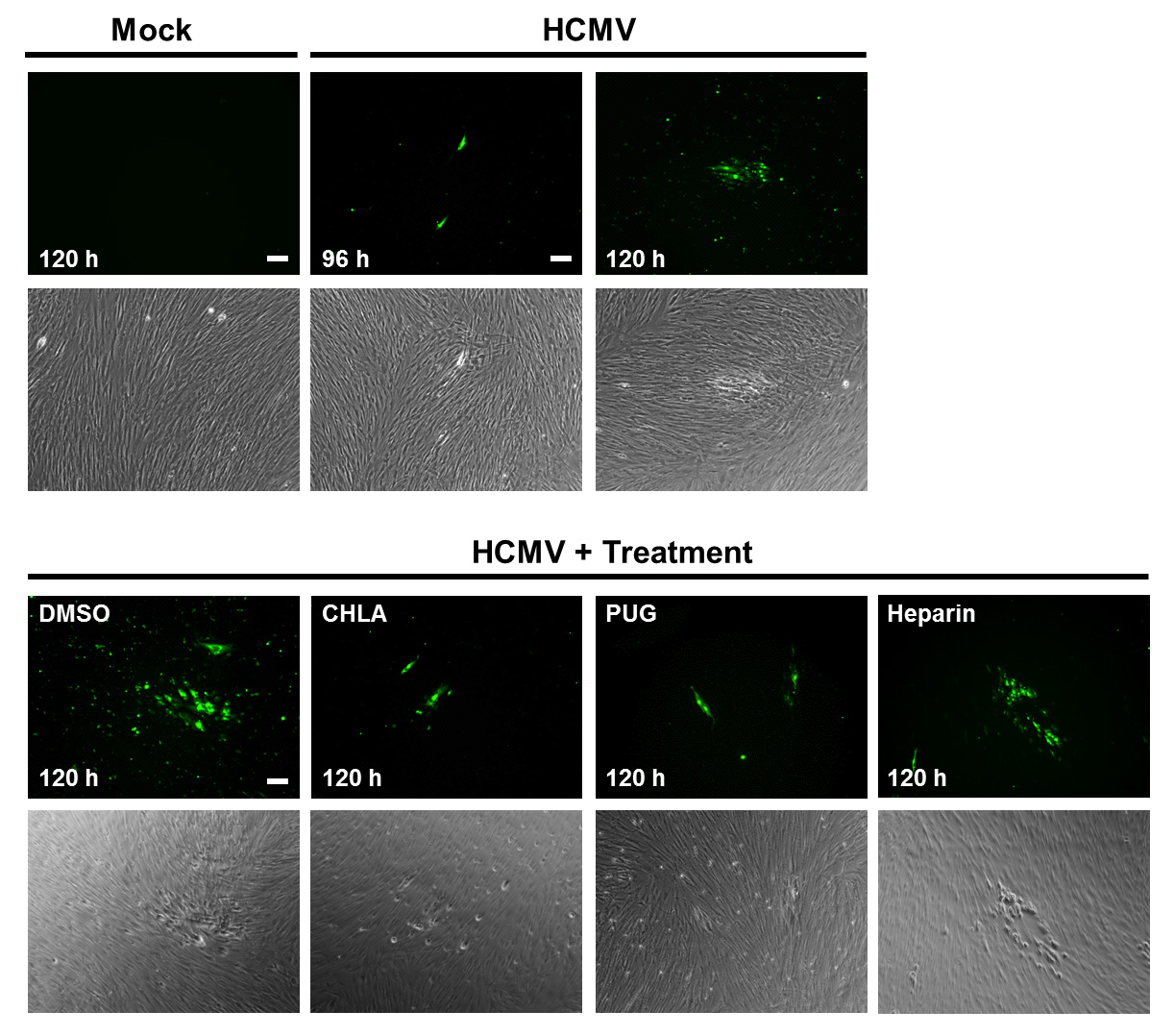

Supplement: Additional file 1: Figure S1 — Examination of CHLA and PUG treatment on HCMV cell-to-cell spread. HEL cell monolayers were inoculated and infected with HCMV for 2 h, washed with PBS to remove excess surface bound virus, and covered with an overlay medium to prevent secondary infection. Initial virus plaques were allowed to form in the subsequent infections and CHLA, PUG, Heparin, DMSO control were added to the overlay medium for an additional incubation time before analysis of viral plaque size by immune fluorescence microscopy at 5 days post-infection as described in Methods. Representative virus plaques/foci are shown after three independent experiments were performed. Scale bar indicates 100 μm. [file 1471-2180-13-187-S1.jpeg]

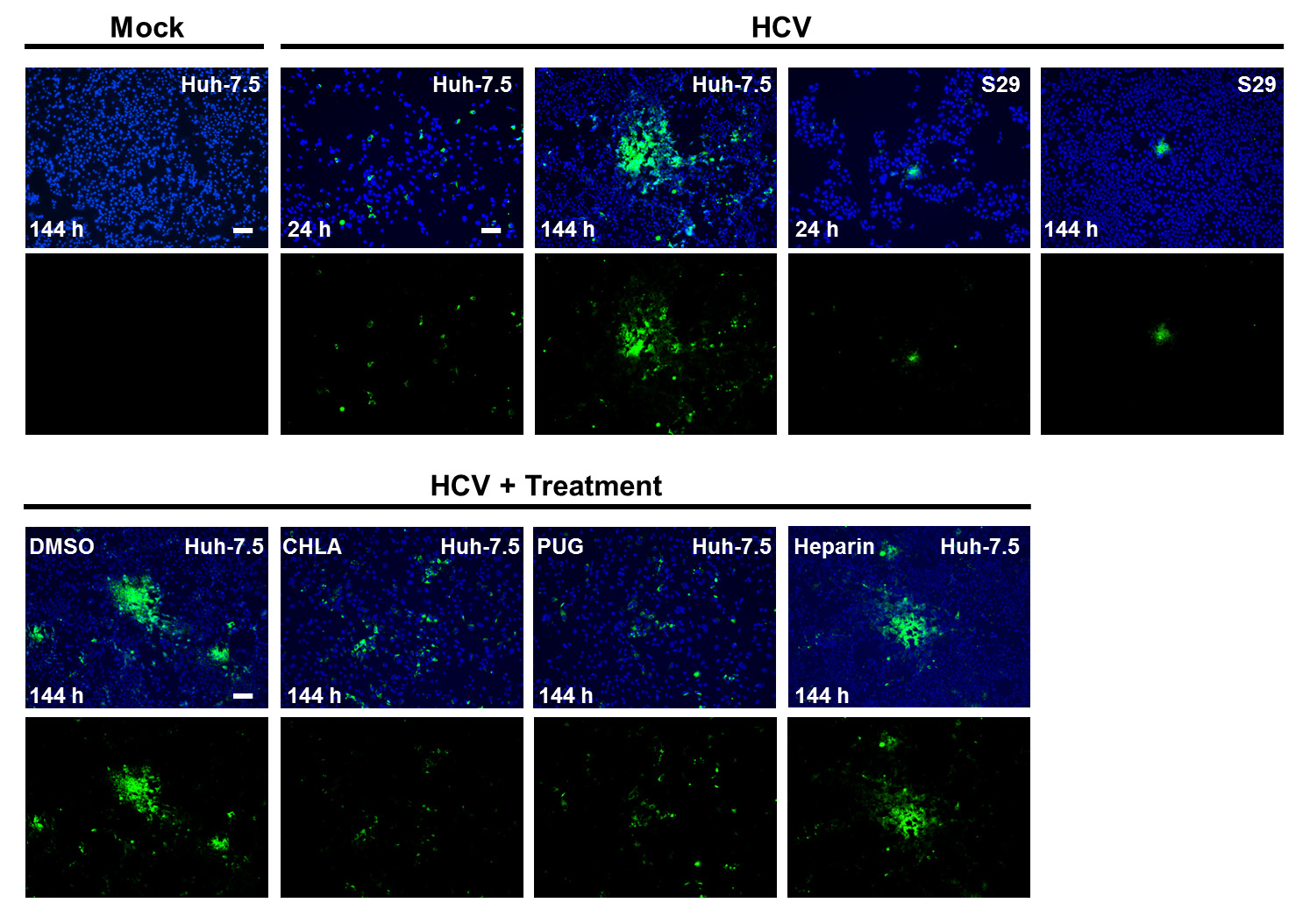

Supplement: Additional file 2: Figure S2 — Examination of CHLA and PUG treatment on HCV cell-to-cell spread. Huh-7.5 cells were electroporated with full-length HCV replicon RNA and covered with an overlay medium to prevent secondary infection. Initial virus plaques were allowed to form in the subsequent infections and CHLA, PUG, Heparin, and DMSO control were added to the overlay medium for an additional incubation time before analysis of viral plaque size by immune fluorescence microscopy at 7 days post-electroporation as described in Methods. Representative virus plaques/foci are shown after three independent experiments were performed. Scale bar indicates 100 μm. [file 1471-2180-13-187-S2.jpeg]

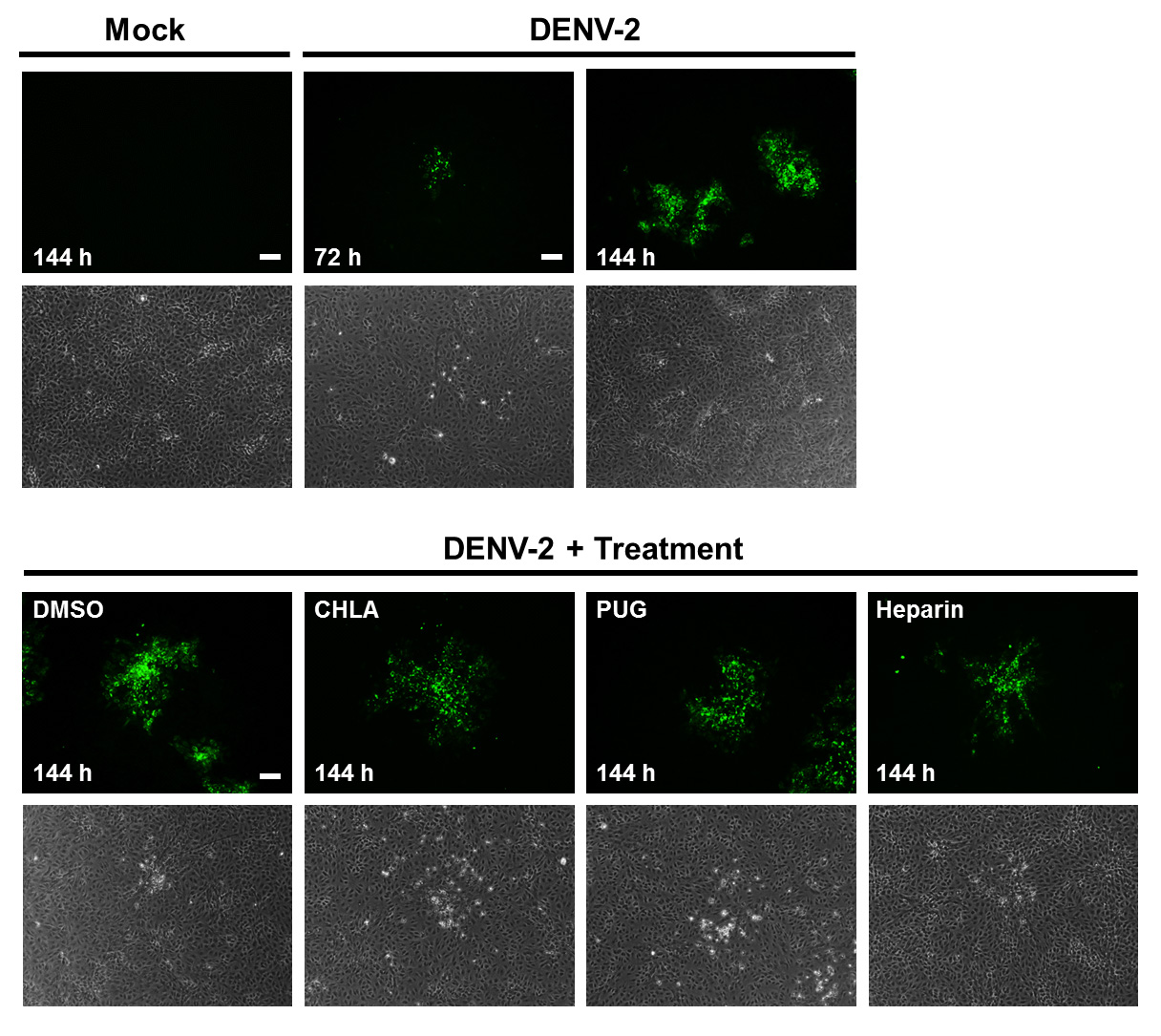

Supplement: Additional file 3: Figure S3 — Examination of CHLA and PUG treatment on DENV-2 cell-to-cell spread. Vero cells were inoculated and infected with DENV-2 for 1.5 h, washed with citrate buffer to remove excess surface bound virus, and covered with an overlay medium to prevent secondary infection. Initial virus plaques were allowed to form in the subsequent infections and CHLA, PUG, Heparin, and DMSO control were added to the overlay medium for an additional incubation time before analysis of viral plaque size by immune fluorescence microscopy at 6 days post-infection as described in Methods. Representative virus plaques/foci are shown after three independent experiments were performed. Scale bar indicates 100 μm. [file 1471-2180-13-187-S3.jpeg]

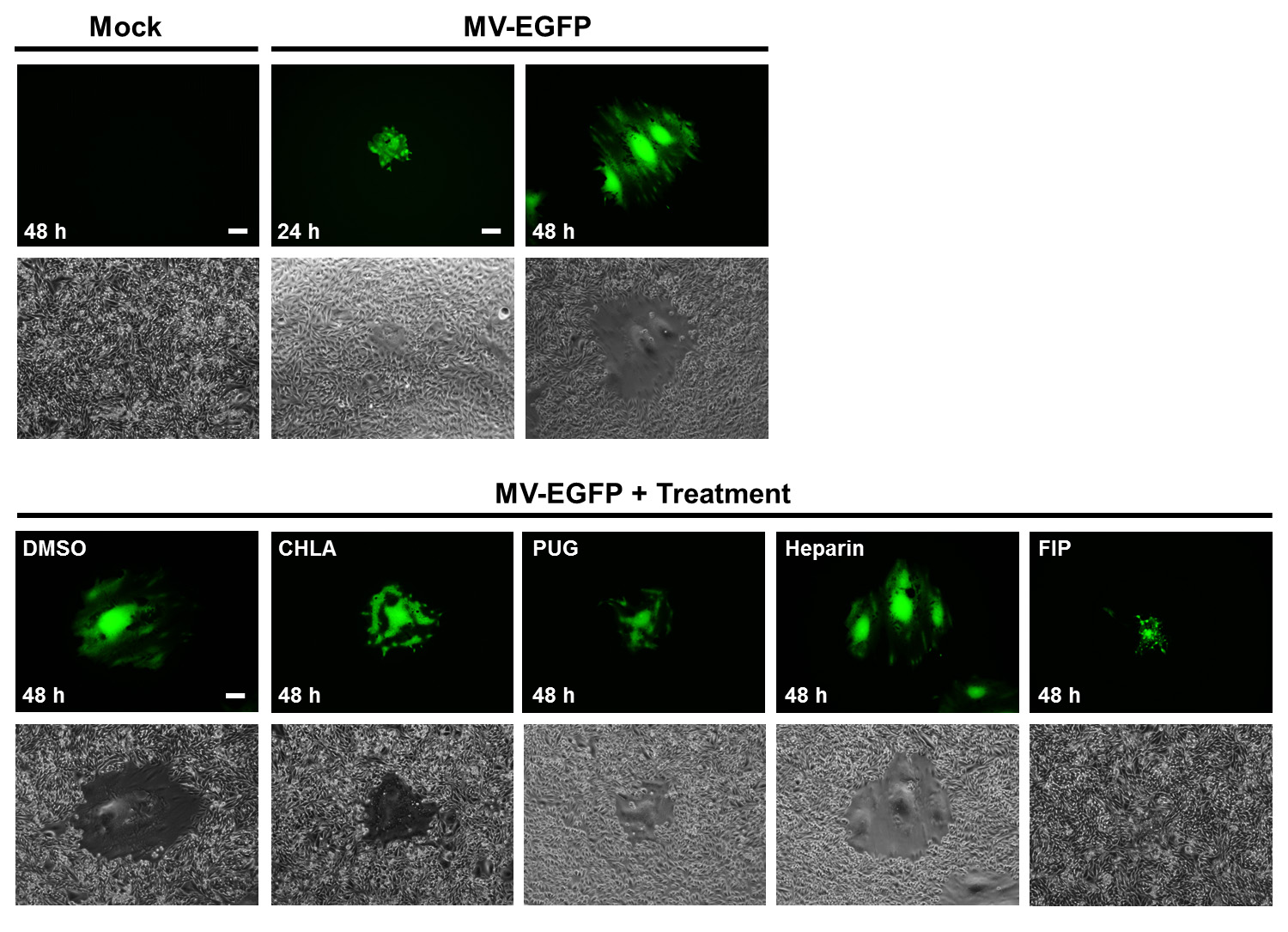

Supplement: Additional file 4: Figure S4 — Examination of CHLA and PUG treatment on MV-EGFP cell-to-cell spread. CHO-SLAM cells were inoculated and infected with MV-EGFP for 1.5 h, washed with citrate buffer to remove excess surface bound virus, and covered with an overlay medium to prevent secondary infection. Initial virus plaques were allowed to form in the subsequent infections and CHLA, PUG, Heparin, FIP, and DMSO control were added to the overlay medium for an additional incubation time before analysis of viral plaque size by EGFP fluorescence microscopy at 48 h post-infection as described in Methods. Representative virus plaques/foci are shown after three independent experiments were performed. Scale bar indicates 100 μm. [file 1471-2180-13-187-S4.jpeg]

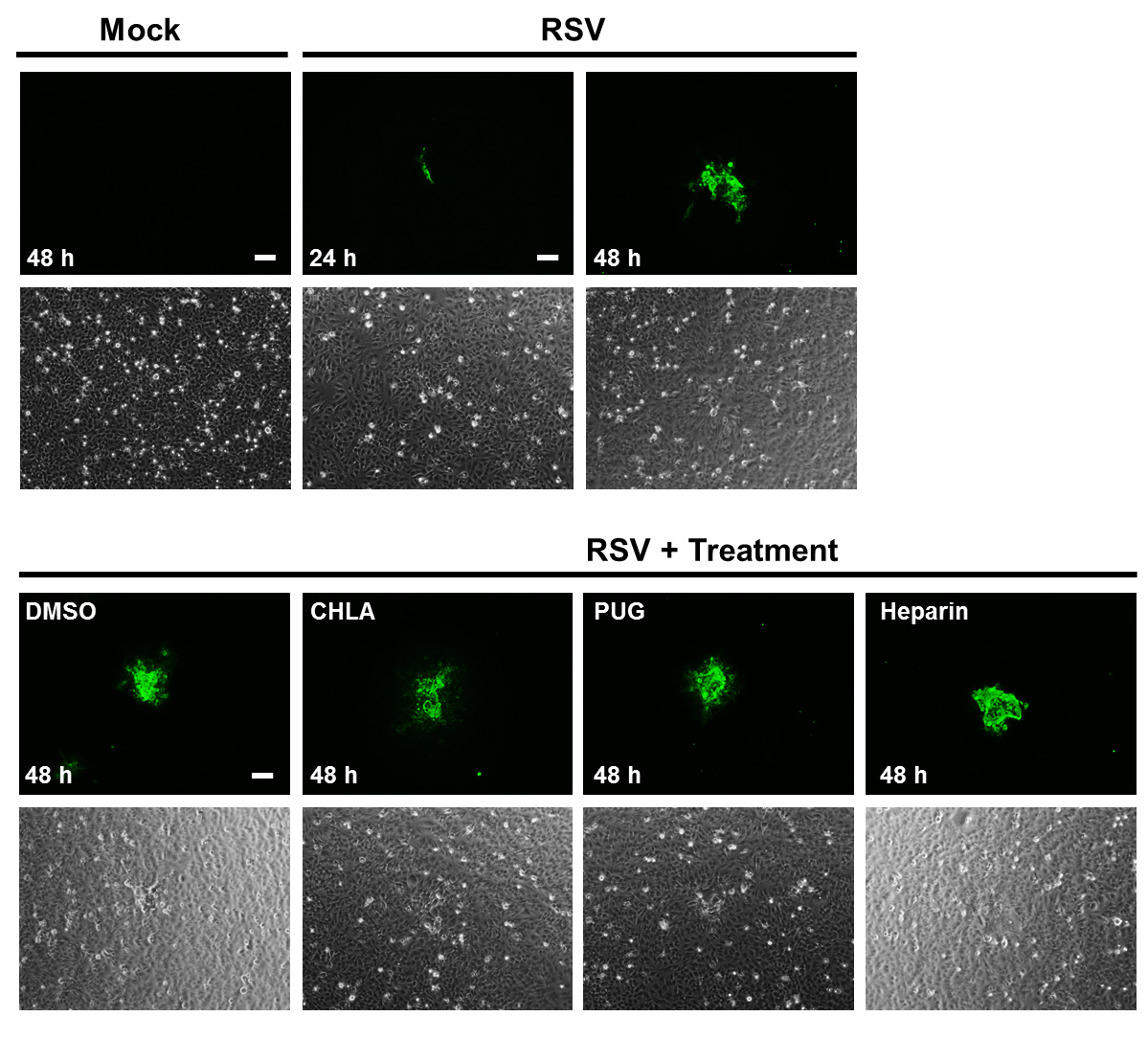

Supplement: Additional file 5: Figure S5 — Examination of CHLA and PUG treatment on RSV cell-to-cell spread. HEp-2 cells were inoculated and infected with RSV for 1.5 h, washed with citrate buffer to remove excess surface bound virus, and covered with an overlay medium to prevent secondary infection. Initial virus plaques were allowed to form in the subsequent infections and CHLA, PUG, Heparin, and DMSO control were added to the overlay medium for an additional incubation time before analysis of viral plaque size by immune fluorescence microscopy at 48 h post-infection as described in Methods. Representative virus plaques/foci are shown after three independent experiments were performed. Scale bar indicates 100 μm. [file 1471-2180-13-187-S5.jpeg]
